# Peptide‐Perovskite Based Bio‐Inspired Materials for Optoelectronics Applications

**DOI:** 10.1002/advs.202408919

**Published:** 2025-01-28

**Authors:** Samrana Kazim, M. P. U. Haris, Shahzada Ahmad

**Affiliations:** ^1^ Materials Physics Center CSIC‐UPV/EHU Paseo Manuel de Lardizabal 5 Donostia‐San Sebastian 20018 Spain; ^2^ BCMaterials Basque Center for Materials Applications, and Nanostructures UPV/EHU Science Park Leioa 48940 Spain; ^3^ IKERBASQUE Basque Foundation for Science Bilbao 48009 Spain; ^4^ Interdisciplinary Research Center for Sustainable Energy Systems (IRC‐SES) King Fahd University of Petroleum and Minerals (KFUPM) Dhahran 31261 Saudi Arabia

**Keywords:** aromatic peptides, biomedical applications, halide perovskite, nanocrystals, short peptides, supramolecular chemistry

## Abstract

The growing demand for environmentally friendly semiconductors that can be tailored and developed easily is compelling researchers and technologists to design inherently bio‐compatible, self‐assembling nanostructures with tunable semiconducting characteristics. Peptide‐based bioinspired materials exhibit a variety of supramolecular morphologies and have the potential to function as organic semiconductors. Such biologically or naturally derived peptides with intrinsic semiconducting characteristics create new opportunities for sustainable biomolecule‐based optoelectronics devices. Affably, halide perovskite nanocrystals are emerging as potentially attractive nano‐electronic analogs, in this vein creating synergies and probing peptide‐perovskite‐based bio‐electronics are of paramount interest. The physical properties and inherent aromatic short‐peptide assemblies that can stabilize, and passivate the defects at surfaces assist in improving the charge transport in halide perovskite devices. This review sheds light on how these peptide‐perovskite nano‐assemblies can be developed for optical sensing, optoelectronics, and imaging for biomedical and healthcare applications. The charge transfer mechanism in peptides along with as an outlook the electron transfer mechanism between perovskite and short peptide chains, which is paramount to facilitate their entry into molecular electronics is discussed. Future aspects, prevailing challenges, and research directions in the field of perovskite‐peptides are also presented

## Graphical abstract

The role of short‐peptide is examined in crystal growth control, as an additive, interfacial agent in lead halide bulk perovskite, as surface ligand in the synthesis of perovskite nanocrystals for solar cells and light emitting diodes applications and future viewpoints on how these peptide‐perovskite nano‐assemblies can be developed for improved performance in optical sensing, optoelectronics, and imaging for biomedical and healthcare applications.

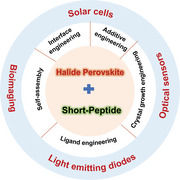

## 1 Introduction

**Figure 1:**
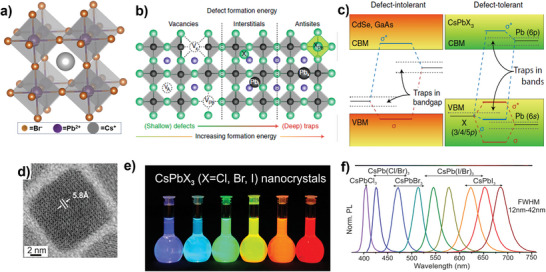
Schematic representation of a) CsPbBr_3_ crystal structure adopts the cubic symmetry and 3D interconnection of PbBr_6_ octahedra. b) typical defects present in the LHP lattice showing vacancies, interstitial, and antisite atoms, in the order of formation energy increases and their location in the bandgap. c) Schematic representation of electronic band structure of defect‐intolerant semiconductors (e.g., CdSe, GaAs) and defect‐tolerant LHP (e.g., CsPbX_3_). In conventional semiconductors, the bandgap is made up of bonding (σ) and antibonding (σ*) orbitals, while in LHP, the bandgap is formed between two antibonding orbitals. As a result, defects will only create shallow traps or be confined inside the valence or conduction band. 1b and c) Reproduced with permission.^[^ ^12^ ^]^ Copyright, 2018, Springer Nature. d) HAADF‐STEM image of a single CsPbBr_3_. e) photographs of colloidal solutions of CsPbX_3_. f) representative PL spectra (λ_exc_  = 400 nm for all but 350 nm for CsPbCl_3_. (d‐f) Reproduced with permission.^[^ ^20^ ^]^ Copyright 2015, American Chemical Society.

Halide perovskites with a general formula ABX_3_ (where, A refers to Cesium {Cs^+^}, methylammonium {MA^+^}, or formamidinium{FA^+^}, while B = mostly Lead {Pb^2+^} or Tin {Sn^2+^} cation and X = chloride {Cl^−^}, Bromide {Br^−^}, Iodide {I^−^}anion)  heralded a new era of photonic and optoelectronic materials, and have emerged as a potential candidate for a broad range of applications such as solar cells,^[^ ^1^ ^]^ light emitting diodes (LEDs),^[^ ^2^ ^]^ photodetectors,^[^ ^3^ ^]^ lasers, sensors,^[^ ^4^ ^]^ memristors,^[^ ^5^ ^]^ etc. **Figure** 1a shows the crystal structure of Cesium Lead bromide (CsPbBr_3_) perovskite, which exhibits cubic symmetry and the PbBr_6_ octahedra expands to a three‐dimensional (3D) network by connecting the corners. From the seminal work of methylammonium lead halides by Weber in 1978^[^ ^6^ ^]^ and the pioneering work by Mitzi in the 1990s,^[^ ^7^, ^8^ ^]^ the use of halide perovskites witnessed unparalleled research and development in solar cells. The performance of lead halide perovskite in thin film solar cells stems from interrelated phenomena, including a direct tuneable band gap with high absorption coefficients,^[^ ^9^ ^]^ low effective carrier masses, high charge carrier mobilities;^[^ ^10^ ^]^ long carrier diffusion lengths; and low excitonic binding energy.^[^ ^11^ ^]^ Halide perovskites entail a cost‐effective fabrication route as compared to the classical traditional semiconductors and have a high tolerance toward defect state and surface without noticeably reducing their performance.^[^ ^12^ ^]^ The likely structural defects in lead halide perovskites are signified by vacancies (mainly A^−^ and X^−^site vacancies) that are depicted by necessarily low formation energies and thus observed typically.^[^ ^13^ ^]^ Nevertheless, these vacancies give rise to localized shallow defect states and reside near or within valence or conduction bands but not within the band gap, unlike conventional semiconductors such as Cadmium Selenide (CdSe), and Gallium Arsenide (GaAs). (Figure 1b,c)

Akin to the thin film, single crystal, and polycrystalline metal halide perovskites, their nanostructures have attracted interest for their quantum confinement effects.^[^ ^14^ ^]^ Nanocrystals of various sizes and shapes from nanoplatelets,^[^ ^15^ ^]^ nanowires,^[^ ^16^ ^]^ and nanorods^[^ ^17^ ^]^ to quantum dots^[^ ^18^ ^]^ can be realized by tuning the synthetic process and conditions. Among the various synthetic techniques, the ligand‐assisted reprecipitation (LARP)^[^ ^19^ ^]^ and hot‐injection (HI) techniques^[^ ^20^ ^]^ led to the high‐quality perovskite nanocrystals (PNCs).

Compared to the bulk halide perovskites, PNCs exhibit unique optical properties that depend on the size distribution, capping ligands, compositions, and reaction temperatures. For example, PNCs bandgap is tunable to cover fully the visible spectrum with a narrow emission peak (full‐width half maxima {FWHM}<100 meV) (Figure 1d,e), the high color purity equates to human color perception and high photoluminescence quantum yields (PLQYs), which can attain the peak values of unity,^[^ ^21^ ^]^ without any electronics passivation at surfaces unlike conventional II‐VI and III‐V semiconductors colloidal nanocrystals, e.g., CdSe & InP respectively. This high photoluminescence quantum yield arises from the defect‐tolerant nature of lead halide perovskites and the electronic structure of the conduction (CB) and valence bands (VB).^[^ ^21^ ^]^

Arguably, PNCs are suitable as lasing material, color converters for lighting and full‐color displays, multicolor emitters in LEDs, and single photon emitters for quantum technology.^[^ ^22^ ^]^ However, compared to bulk perovskites (3D), the charge transport characteristics of PNCs lag. One of the key problems that avoids the use of PNCs for specific electrical applications is the electron‐hole Coulomb interactions in effective screening as compared to bulk materials. In contrast to bulk perovskites, nanoparticles bounded by an organic media have a low value of dielectric constant with exciton binding energies in the range of 200−500 meV that are up to an order of magnitude higher than bulk counterparts. Thus, the higher exciton binding energy in PNCs significantly reduces the exciton dissociation probability, before radiative decay as compared to the bulk, which results in a higher PLQY and poor charge transfer. Moreover, PNC's high sensitivity to polar solvents or instability makes them incompatible with biological applications that frequently use polar media like water.

On the other hand, the growing demand for eco‐friendly organic semiconductors that can be easily developed and tailored is of significant interest for researchers to design inherently bio‐inspired, self‐assembling peptides and protein‐based nanostructures with enhanced semiconducting features. Among them, short aromatic peptides are the most favored building blocks due to their self‐assembly merits as flexible supramolecular structures with distinctive physical and chemical characteristics, including attractive semiconducting properties. Such bioinspired materials can potentially not only function as organic semiconductors but can also synchronize the gap between the biological and semiconductor fields to transform bio‐electronics and healthcare applications.

**Figure 2:**
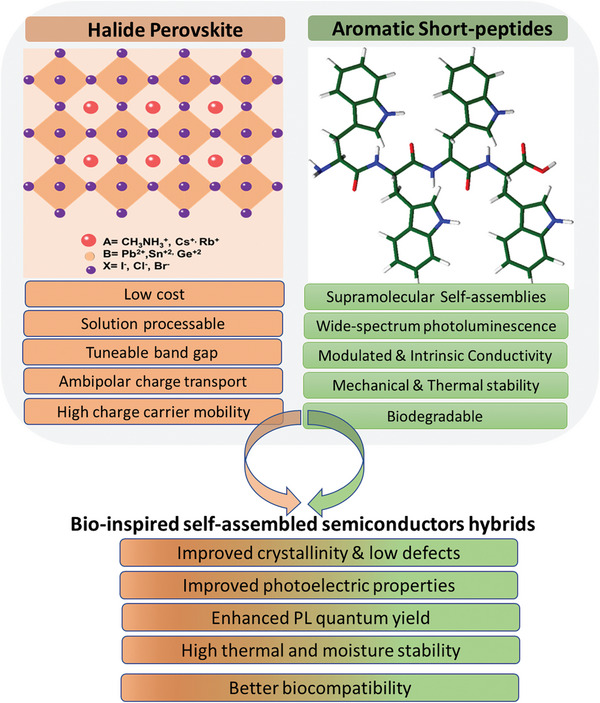
Summary of advantages of halide perovskite and short peptide materials & their Improvement of material properties by combining perovskite‐peptide.

Short peptide‐regulated self‐assemblies with photo‐active moieties^[^ ^23^, ^24^, ^25^ ^]^ have been investigated for the construction of biomimetic systems for photosynthetic reactors^[^ ^26^ ^]^ and anti‐cancer therapy,^[^ ^27^ ^]^ which were inspired by protein‐based natural systems.^[^ ^28^ ^]^ They have been probed for their possible applications in optical sensing, imaging, photocatalysis,^[^ ^29^ ^]^ ferroelectric‐based devices and are future candidates as functional nanostructures due to their structural variety and cost‐effective synthesis. The interest in the design principles of supramolecular materials based on aromatic short‐peptides for scientific innovation is huge. These aromatic short peptides self‐assemblies have remarkable micromechanical toughness and thermal stability, notable photoluminescence in wide spectral range, and thermo‐ and opto‐modulated conductivity due to the extensive and directional hydrogen bonding and aromatic interactions. Thus, creating synergies between short‐peptides and lead‐free halide perovskites could be a game changer in bridging the perovskite optoelectronics and bio‐medical applications (**Figure** **2**).

**Figure 3:**
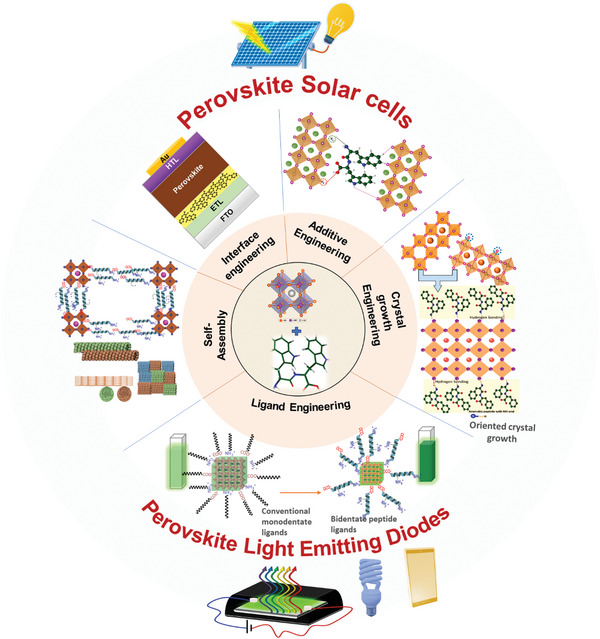
Potential applications of short‐peptide explored in self‐assembly process and perovskite optoelectronics.

This review sheds light on the self‐assemblies and optoelectronic properties of short‐peptides and uncover the underlying mechanism of their optoelectronic behavior. Besides, their applicability in the synthesis of lead halide PNCs for their different roles such as capping ligands for colloidal stability, controlling the crystal growth, as an additive, interfacial agent in lead halide perovskite optoelectronics devices, have been elaborated (**Figure** **3**). Further, we discussed their effects on environmental stability against atmospheric stress factors for optoelectronics applications along with the potential of using aromatic short peptides in core‐shell nanoparticles, as charge‐transporting materials. This can be further conjugated with organic semiconductors, as an encapsulator, or A‐site aromatic spacer cation in layered perovskite.

## 2 Self‐Assemblies and Optoelectronic Properties of Aromatic Short‐Peptides and their Underlying Mechanism

**Figure 4:**
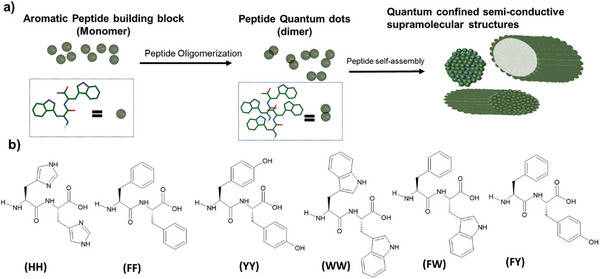
a) Peptide self‐assembling architectures show intrinsic semiconductive properties. Peptide building blocks oligomerize to quantum dots, which then self‐assemble into supramolecular structures with diverse morphologies to serve as bioinspired organic semiconductors. Adapted with Permission.^[^ ^30^ ^]^ Copyright 2017, AAAS. b) Examples of a few aromatic dipeptide materials that can be used for optoelectronics applications with perovskites.

Supramolecular self‐assembly of short peptides offers exciting opportunities for creating functional materials at the molecular to nano‐ and micrometer scale. Peptides are organic materials composed of two or more amino acids that bind together through peptide bonds and are typical building blocks of bio‐molecules. Their ease of synthesis, diversity in structure, cost‐effectiveness, and simplicity of functionalization make them an alternative to today's optoelectronic devices based on conventional inorganic semiconductors.^[^ ^30^, ^31^ ^]^ These building blocks can be organized either linearly (linear peptide) or in a cyclic ring (cyclic peptide) which can be self‐assembled into various well‐ordered nanostructures and supramolecular morphologies (**Figure** **4**a) and their properties can be simply tailored.

Recent research has discovered that certain naturally occurring polypeptide and protein self‐assemblies exhibit intrinsic semiconductive capabilities^[^ ^32^ ^]^ and attractive photoluminescence properties, which are induced by hydrogen bonding networks between peptide backbones^[^ ^33^ ^]^ or π‐π aromatic conjugation interactions between side‐chain aromatic moieties.^[^ ^34^ ^]^ Similar to nature‐derived peptides and proteins, aromatic short peptides also consist of hydrogen bonds and aromatic interactions, and are the most extensively researched short peptides that self‐assemble into a wide range of supramolecular semiconductive nano‐structures that are kinetically or thermodynamically stable.^[^ ^35^ ^]^

The illustrative model of the aromatic short peptide is diphenylalanine (FF), which produces numerous self‐assembling semiconductive architectures. The other examples of aromatic short dipeptides are tryptophan (WW), tyrosine (YY), or combinations of these amino acids in different peptide sequences, for instance, phenylalanine‐tryptophan (FW) or phenylalanine‐ tyrosine (FY). (Figure 4b) All these aromatic amino acids were noted to assemble into amyloid‐like structures, exhibiting intrinsic fluorescence in the blue region (450 nm). Among them, Tryptophan shows high quantum yield and emission at longer wavelengths compared with the other three aromatic amino acids.^[^ ^36^ ^]^

These short peptides exhibit optical and electric properties due to quantum‐confined structures inside the self‐assemblies that are comparable to those of traditional inorganic semiconductors.^[^ ^35^ ^]^ The strong Coulomb interaction between highly confined electrons and holes produces structure‐dependent optical properties and can lead to the formation of an exciton with specific absorption and luminescence.^[^ ^37^, ^38^ ^]^ To illustrate, the spike‐like patterns in the absorbance spectra of FF nanotubes^[^ ^34^ ^]^ and tert‐butyloxycarbonyl protected diphenylalanine (Boc‐FF) nanospheres^[^ ^39^ ^]^ demonstrate the presence of 0D QDs in the assemblies. On the other hand, the absorption spectra of 9‐fluorenylmethoxycarbonyl (Fmoc) protected diphenylalanine (Fmoc‐FF) nanofibrillar hydrogels display a distinct step‐like absorption pattern and a peak at the long‐wavelength edge produced from the increased Coulomb interaction of excitons‐ which is a characteristic of 2D quantum well confinement structures.^[^ ^37^ ^]^

Several approaches have been used to investigate and facilitate the self‐assembly of FF and its derivatives. One of the methods describes the use of various solvents, in which FF can self‐assemble into unique nanostructures and exhibit distinct optical properties. Other approaches produce self‐assembled FF with adjustable morphologies and improved semiconductivity through the incorporation of external semiconductive subunits such as fullerene derivatives or perylene imide moieties.^[^ ^24^, ^25^, ^40^, ^41^ ^]^

Thus, the pre‐requisite for short peptide self‐assembling semiconductors is that the driving forces, including π‐π aromatic conjugated interactions and hydrogen bonding, should be able to reduce the band gaps down to the semiconductive region. The extension of π‐π conjugation along the peptide backbone can delocalize the electrons over a larger molecular orbital. This can minimize the energy difference between the highest occupied molecular orbital (HOMO) and lowest unoccupied molecular orbital (LUMO) orbitals. On the other side, the hydrogen bonding can hold specific peptide conformations which favors the extension of π‐π conjugation and facilitate the bandgap reduction. Further, the semiconductivity of the superstructures can be tuned by controlling the extent of driving forces. Moreover, the material´s morphology significantly impacts the semiconductive characteristics, therefore, the nanostructures created by the self‐assembly of short‐peptide building blocks can allow fine‐tuning of semiconductive characteristics.

Their band gap can be tuned by the substitution of amino acids or side aromatic group moieties. Tryptophan (Trp)‐based aromatic dipeptide self‐assemblies are projected to integrate and enhance physicochemical properties in a single system,^[^ ^42^ ^]^ because of the additional hydrogen bonds and π‐π aromatic interactions mediated by the side‐chain heterocyclic indole ring. Thus, the increased number of hydrogen bonding and aromatic interaction networks can decrease the bandgap between the HOMO and LUMO levels.^[^ ^43^, ^44^ ^]^ For instance, upon substitution of one F in diphenylalanine (FF) with tryptophan (W), self‐assembling phenylalanine‐tryptophan (FW) nanostructures present a smaller bandgap of 2.25 eV, compared to 3.25 eV of FF assemblies,^[^ ^45^ ^]^ due to increased aromatic π‐π interactions and denser molecular interstitial region, which in turn shows improved conductivity by a factor of 5 relative to FF nanotubes^[^ ^46^ ^]^ and photoluminescence properties.^[^ ^47^ ^]^

**Figure 5:**
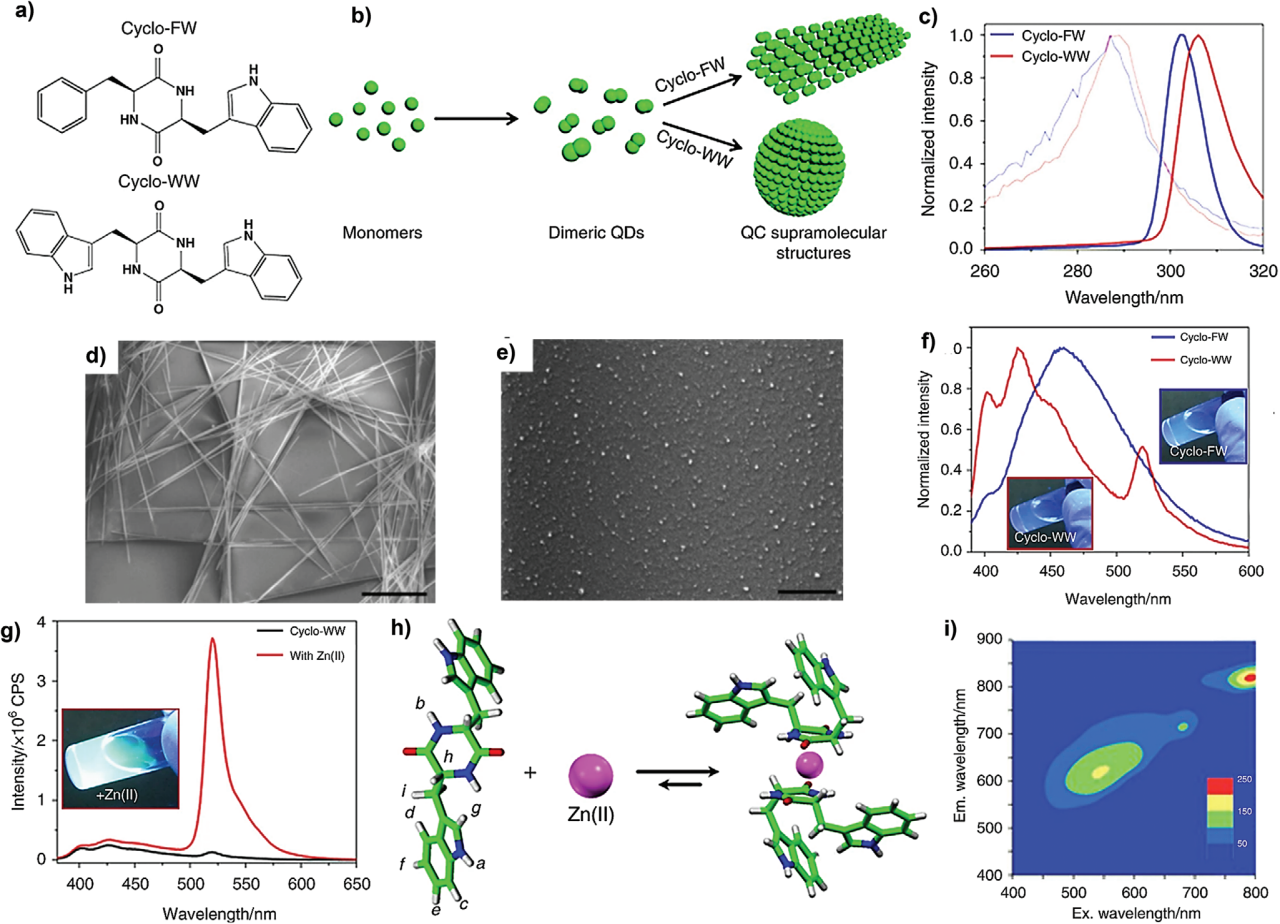
Optoelectronic properties of the cyclo‐dipeptide crystals. a) Molecular structures of cyclo‐FW and cyclo‐WW. b) Schematic representing the process of cyclo‐dipeptides self‐assembly: the monomers form dimeric QDs, which serve as the building blocks to self‐assemble into larger QC architectures. c) Excitation spectra of cyclo‐dipeptide monomers (thin light curves, 0.05 mM) and self‐assemblies (thick dark curves, 5.0 mM) in MeOH. The excitation wavelengths red‐shifted from 285 nm to ≈305 nm after self‐assembly. d‐e) SEM images of the cyclo‐dipeptides QC self‐assemblies in MeOH. d) Needle‐like cyclo‐FW crystals., Scale bar: 40 µm. e) Spherical cycloWW nanoparticles, Scale bar: 20 µm. f) Fluorescent emission of cyclo‐FW(blue) and cyclo‐WW (red) self‐assemblies. g) Fluorescent emission of cyclo‐WW + Zn(II). h) Schematic presentation showing the possible molecular mechanism of cyclo‐WW dimer coordination with Zn(II): the backbone diketopiperazine rings contribute to the complexation through nitrogen atoms, while the side‐chain indole rings form aromatic interactions and i) Emission versus excitation profile of the nanospheres. Reproduced under the terms of CC‐BY‐4.0.^[^ ^49^ ^]^ Copyright 2018, The Authors, published by Springer Nature.

Besides linear aromatic peptides, another intriguing family of peptides that have drawn a lot of attention in the modulation of optical properties are cyclic peptides. Peptides in the cyclic form have optoelectronic properties that are superior to their linear counterparts due to their structural stiffness, unique hydrogen bonding style, stronger intermolecular interactions caused by the existence of two peptide bonds, and greater proteolytic stability. Tryptophan‐based aromatic cyclo‐dipeptides, such as cyclo‐FW and cyclo‐WW (**Figure** **5**a) tend to form dimeric quantum dots (Figure 5b)^[^ ^48^, ^49^ ^]^ exhibited tunable photoluminescence properties from the formation of a quantum‐confined assembly, which is further endorsed by a red shifting of the molecular excitation to 305 nm compared to 285 nm for the monomers in Fluorescent excitation measurements (Figure 5c), suggesting that the cyclo‐dipeptides are self‐assembled into different supramolecular morphologies (Figure 5d‐e). Upon excitation at 370 nm wavelength, the cyclo‐dipeptides solutions demonstrated emission in the visible region at 460 nm for cyclo‐FW and 425 nm for cyclo‐WW along with a side band at 520 nm (Figure 5f). A shoulder peak at 529 nm (excited at 300 nm) indicates the presence of the intrinsic emission of amino acid residues.^[^ ^49^ ^]^

The tunable optical properties can be achieved by amino acids substitutions and sequence,^[^ ^47^ ^]^ solvent replacement, metal ion coordination,^[^ ^47^ ^]^ covalent conjugation^[^ ^50^ ^]^ or co‐assembly with external moieties^[^ ^51^ ^]^ oxidization of the molecules,^[^ ^52^ ^]^ or irradiation under different wavelengths of UV, all of them directly impacts on the dipeptides's self‐assembled structures ranging from dimeric QDs to supramolecular organizations.^[^ ^42^ ^]^ Doping is found to be a successful method for improving semiconductors' characteristics.^[^ ^53^ ^]^ Cyclo Tryptophan‐Tryptophan (WW) self‐assembling nanoparticles (NPs) exhibit luminous green fluorescence at 520 nm (Figure 5g) when coordinated with Zinc (Zn^2+^) ions (Figure 5h) and quantum yield is estimated to be ≈16%.^[^ ^49^ ^]^ Moreover, Fluorescent emission can be adjusted to cover a broad spectrum from the visible to near‐infrared spectral regions using a wide range of excitation wavelengths (Figure 5i).

While cyclo‐HH coordinated with Zn^2+^ demonstrated a 70% quantum yield of bright green fluorescence emission, which is the highest value documented for short peptide‐derived materials to date, utilized as an emissive layer for prototype light‐emitting diodes and as a nanocarrier for intracellular drug delivery and in vivo imaging.^[^ ^54^ ^]^ While the Zn^2+^ coordination stiffens the chromophore and decreases fluorophore mobility, the improved fluorescence was thought to be caused by the self‐assembly locking approach.

For optoelectronic applications, these bioinspired materials may eventually serve as a substitute for their inorganic equivalents.^[^ ^55^ ^]^

## 3 Electron Transfer Mechanism in Peptides

The proteins and peptides are composed of an array of amino acids that play central roles in the natural respiration and photosynthesis processes and are used as a medium for electron transfer reaction that takes place either in single‐step electron tunneling (super ‐exchange) or in multi‐step electron hopping processes. A deeper understanding of the electron transfer kinetics of peptides is critical to realizing the bio‐applicability of PNCs. We need to uncover the various energy transfer processes along the peptides/peptide self‐assemblies and the one between peptide moiety and perovskite or electrode. Through various theoretical and experimental studies, complex conduction phenomena such as electron tunneling (super‐exchange) and electron hopping (sequential pathway) are established as the typical electron transfer mechanisms.^[^ ^55^, ^56^ ^]^

**Figure 6:**
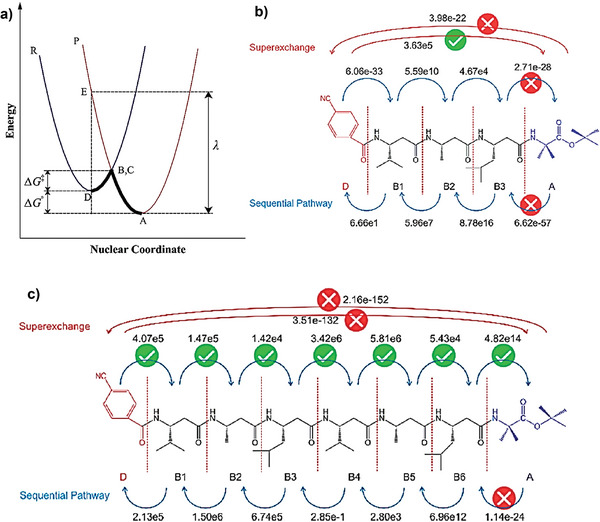
a) Energy diagram for the electron tunneling process, Reproduced with permission.^[^ ^62^ ^]^ Copyright 2021, Elsevier. b,c) electron transfer pathways for b) short‐peptides and c) long peptides (red: tunneling; blue: sequential hopping) with computed transfer rates (in s^−1^) for individual steps, Reproduced with permission.^[^ ^63^ ^]^ Copyright, 2018 American Chemical Society.

Between these two mechanisms, the super‐exchange is highly length‐dependent, and the rate of single‐step electron transfer intensely decreases with increasing the distance, while a hopping process is less distance‐dependent. This is valid only if the side chain in the amino‐acids group contains an oxidizable group, which acts as an intermediate charge carriers, such as phenol in tyrosine. In the hopping process, fast electron transfer over large distances takes place in multistep reactions, as in the enzyme ribonucleotide reductase. Electron tunneling theory explains the electron transfer from an electron donor (D) to an electron acceptor (A) through a connected bridge (B). **Figure** **6**a shows the energy curves for the electron tunneling process, where, the electron transfer from reactant (R) to product (P) occurs only when they have equal nuclear coordinates and energies, represented by the coincidence of bridging points B (surface of R) and C (surface of A). In short, electron tunneling is the transfer process from B to C where the electron is transferred via a virtual bridge connected between donor D and acceptor A.^[^ ^57^, ^58^ ^]^ From the electron transfer rate, it was shown that the transfer rate decays by a factor of 10 with each 2.3 A° increase in distance between points D and A.^[^ ^59^ ^]^ Indicating that the electron tunneling mechanism is suited for short‐peptides and it does not enlighten for long‐range peptides. On the other hand, the electron hopping mechanism, in which the electron transfers occur in several steps via a large number of bridging sites, explains the electron transfer in a long‐range peptide or proteins.^[^ ^60^ ^]^ With the use of electron pulse radiolysis and femtosecond transient absorption spectroscopic techniques, it is deduced that the change from an electron tunneling mechanism to a hopping mechanism when the peptide spacer distance increased from 8.7 to 32 A°.^[^ ^61^ ^]^ Figure 6b,c demonstrate the electron transfer mechanisms in short‐ and long‐range peptides along with their computed rates. However, an in‐depth understanding of the electron transfer between peptide ligand and perovskite or electrode surface is of paramount interest.

## 4 Short‐Peptides in ABX_3_ Perovskites‐Based Optoelectronics

Several research groups have developed a variety of applications utilizing the linear amino‐acids, aliphatic, and aromatic short‐peptide systems due to the short length, straightforward synthesis of the short peptide building blocks, effective and repeatable assembly processes, and special physical features. We discuss the most significant and recent advances in the synthesis and development of halide perovskite crystals and optoelectronics devices using peptides or peptide‐like molecules. We highlight the key advantages of using multifunctional peptide‐like molecules as a capping ligand and passivator for nano and bulk perovskite crystal surfaces and at the interface in the solar cells and light‐emitting diodes as tabulated in **Table** **1**.

**Table 1:** Summary of the various short‐peptide and their role in the treatment of halide perovskite for performance enhancement.

Perovskite	Peptide used	Role of Peptide	Output	Ref
MAPbBr_3_, CsPbBr_3_	12‐AA, 8‐AA, 6‐AA	Surface Ligand in PNC	high crystallinity, uniform size distribution, and high product yield	[78]
MAPbBr_3_	Cyclo‐RGDFK	Surface Ligand in PNC	PLQY = 20%	[79]
MAPbBr_3_	L‐cyst	Surface Ligand in PNC	long‐term structural and chemical stability in protic solvents, high PLQY	[80]
MAPbBr_3_	L‐lys, L‐arg	Surface Ligand in PNC	PLQY of ≈100%	[81]
CsPbBr_3_	BDGA	post‐treatment surface passivation	PLQY of ≈100%, WLEDs with a luminous efficiency of 93.5 lm W^−1^	[77]
MAPbBr_3_	PNA‐M, PNA‐T	surface ligand in PNC	PL quenching, nucleic acid sensing	[87]
MAPbI_3_	(LL)	post‐treatment surface passivation	Enhanced crystallinity, efficient charge transfer, lowered trap state density at the interface, Improved PCE‐18.2%,	[104]
MAPbI_3_	4‐ABPACl	Additive in Perovskite for PSC	Hydrophobic perovskite surface, PCE = 16.7%	[107]
MAPbI_3_	F‐GLU‐S	Additive in Perovskite for PSC	Hydrophobic perovskite surface, *V*_OC_ and FF enhancement, PCE = 21.44%	[108]
Cs_0.05_FA_0.81_MA_0.14_PbI_2.55_Br_0.45_	D4TBP	Perovskite‐HTL interface passivation	Enhanced PCE of 21.4% with reduced *V*_OC_ deficit	[103]
MAPbI_3_	Arg	Perovskite‐HTL interface passivation	*V*_OC_ enhancement and PCE = 20.49%	[100]
MAPbI_3_	l‐ala	TiO_2_‐perovskite interface passivation	30% PCE improvement, Reduced charge transfer resistance at the TiO_2_/CH_3_NH_3_PbI_3_ interface	[99]
MAPbI_3_	L‐cyst	TiO_2_‐perovskite interface passivation	PCE increased from 11.5 to 14.4%	[109]

12‐AA (12‐aminododecanoic acid), 8‐AA (8‐aminododecanoic acid), 6‐AA (6‐aminododecanoic acid), Cyclo‐RGDFK (Cyclo(Arg‐Gly‐Asp‐D‐Phe‐Lys)), L‐cyst(L‐Cysteine amino acid), L‐lys (L‐lysine), L‐arg (L‐arginine), BDGA(Boc‐D‐Glutamic acid), PNA‐M(Peptide Nucleic Acid‐Monomer), PNA‐T(Peptide Nucleic Acid‐trimer), LL(L‐Leucine, (4‐ABPACl (butylphosphonic acid 4‐ammonium chloride), D4TBP (D‐4‐tert‐butylphenylalanine), Arg (arginine), F‐GLU‐S (sulfonyl‐γ‐AA peptide), l‐ala (l‐alanine).

### 4.1 Short‐Peptides in Colloidal PNCs

The surface ligands are indispensable for the synthesis, growth, and colloidal stability of nanocrystals. Due to the soft ionic nature of lead halide perovskite, it favors ionic surface ligands, which show dynamic binding to the surface of PNCs and tend to come off the surface when washing, or aging the colloidal nanocrystals. Furthermore, these processes cause surface atoms to dissolve or degrade, resulting in a disordered surface with vacancy formation at the A and X sites that causes spectral shifts and lowers the PLQY and chemical and thermal stability.^[^ ^64^ ^]^

**Figure 7:**
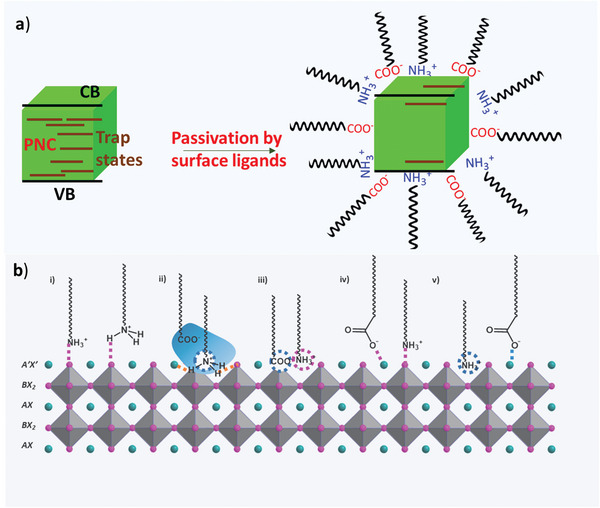
Schematic illustration of the a) elimination of bandgap trap states by Surface passivation with ligands in perovskite NC. b) different binding modes of oleylamine and oleic acid to a lead halide perovskite NC surface. Reproduced under the terms of CC‐ BY‐4.0.^[^ ^75^ ^]^ Copyright 2023, The Authors, published by American Chemical Society.

Despite the unique tuneable optoelectronic properties, the high surface‐to‐volume ratio, the high reactivity of surface atoms, and the non‐stoichiometric elemental ratio at surfaces destabilize the ABX_3_ PNCs.^[^ ^65^, ^66^ ^]^ These uncoordinated surface atoms or ions generate surface defects and sub‐bandgap states which can capture photoexcited electrons and thereby result in non‐radiative recombinations or photoluminescence quenching. The surface defects can be associated with i) organic/inorganic mono‐cation (A‐site cation), and ii) divalent metal cation (B‐site cation) and halide anions (X‐site anion). For example, a (Br) bromine‐rich and (Pb) lead‐poor surface has been reported for methyl ammonium lead bromide nanocrystals where the Br^−^/Pb^2+^ ratio was found as 3.5 at the surface and 3 in the bulk respectively.^[^ ^67^ ^]^ Passivation of surface defects with molecular capping ligands has been suggested as an effective tool to protect and stabilize the PNCs and improve the photoluminescence, this increases quantum yield by eliminating the surface and band gap defect state (**Figure** **7**a).^[^ ^68^, ^69^, ^70^, ^71^, ^72^ ^]^

Ideally, a combination of positive and negatively charged ligand ends is required to passivate the opposite‐charged surface defects (anionic and cationic defects). Various capping ligand combinations such as octylamine with HBr_,_^[^ ^73^ ^]^ oleic acid with octylamine,^[^ ^19^ ^]^ oleic acid with oleylamine,^[^ ^74^ ^]^ etc. have been employed. Among others, Oleic acid (OA) and Oleylamine (OAm) are the most commonly used ligand combinations to synthesize halide PNCs. The acid−base pair ligands first react to each other through proton transfer from the carboxylic acid (R−COOH) to alkyl amine (R−NH_2_) base and convert into a carboxylate anion (R−COO^−^) and ammonium cation(R−NH_3_^+^), (Equation 1) and further, these ions are expected to bind to the surface of lead halide PNCs and can passivate cationic (A^+^) and anionic (X^–^) defects respectively, and well‐passivated and colloidally stable PNCs dispersion in non‐polar solvents is formed. OA and OAm ligand passivated PNC system^[^ ^75^ ^]^ which is schematically shown in Figure 7b.

(1) $$R-COOH+R^{\prime }-NH_{2}↔R-COO^{-}+R^{\prime }-NH_{3}^{+}$$

The structural and optical characteristics of the halide PNCs can be adjusted by adding or removing OA and OAm. Moreover, OA and OAm control nanocrystal self‐assembly to produce well‐organized thin films. Additionally, the charge transport characteristics are significantly influenced by these ligands's presence on the nanocrystal surface. However, due to their large size, these ligands exhibit significant surface‐capping capabilities, and can significantly hamper charge transport in nanocrystal films due to their insulating nature. Typically in chalcogenide QDs for exchange, ligands with shorter lengths are preferred with halide PNCs to increase charge carrier mobility and thus optoelectronic performance.^[^ ^76^ ^]^ Further, the surface functionalization with rational ligand molecules could impart environmental stability against external stresses along the passivation of surface traps and add new functionalities.

The non‐covalent interactions present in self‐assembled peptides show their dynamic bonding nature, which can link with well‐established PNCs. Currently, the protocol for stabilizing the PNCs uses separate ligands with carboxylic and amino end groups. Instead of using separate ligands for anionic and cationic defect sites, a single multifunctional ligand will simplify the synthesis. In this context, the peptide compounds containing both carboxylic and amino groups demonstrated their ability to serve as an alternative. In addition to their use as single multidentate ligand‐assisted PNC syntheses, the use of peptides as capping ligands can extrapolate the PNC applicability to biological devices. Similar to organic ligand passivation, the short peptide with ─COOH and ─NH_2_ group can self‐assemble at the surfaces and grain boundaries to passivate the under‐coordinate Pb^2+^ and halide vacancies. Thus quality films can be expected with improved PL emission and carrier lifetime.^[^ ^77^ ^]^

**Figure 8:**
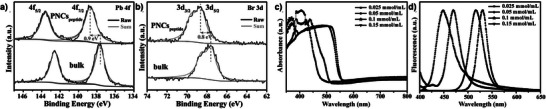
a,b) XPS peak shift to higher binding energy by peptide passivation for a) Pb 4f and b) Br 3d core levels. c) UV‐Vis absorption. and d) PL peak shifting with varying ligand concentration. Reproduced with permission.^[^ ^78^ ^]^ Copyright 2017, John Wiley and Sons.

In preliminary work, the employment of different peptide‐like molecules as capping ligands such as 12‐amino dodecanoic acid (12‐AA), 8‐amino octanoic acid (8‐AA), and 6‐amino hexanoic acid (6‐AA) in PNC synthesis through a LARP method is shown.^[^ ^78^ ^]^ The attained high crystallinity, uniform size distribution, and high product yield substantiated their hypothesis for the adaptability of peptides as capping ligands. As depicted (**Figure** **8**a,b) they observed a shift to higher binding energy for Pb 4f and Br 3d core level spectra in X‐ray photoelectron spectroscopy with peptide capping. This strengthened the objective of peptide capping to passivate the surface defects. Akin to the other capping ligands, peptide ligands display concentration‐dependent size distribution, and shifts in the corresponding absorption and emission profiles (Figure 8c,d).

**Figure 9:**
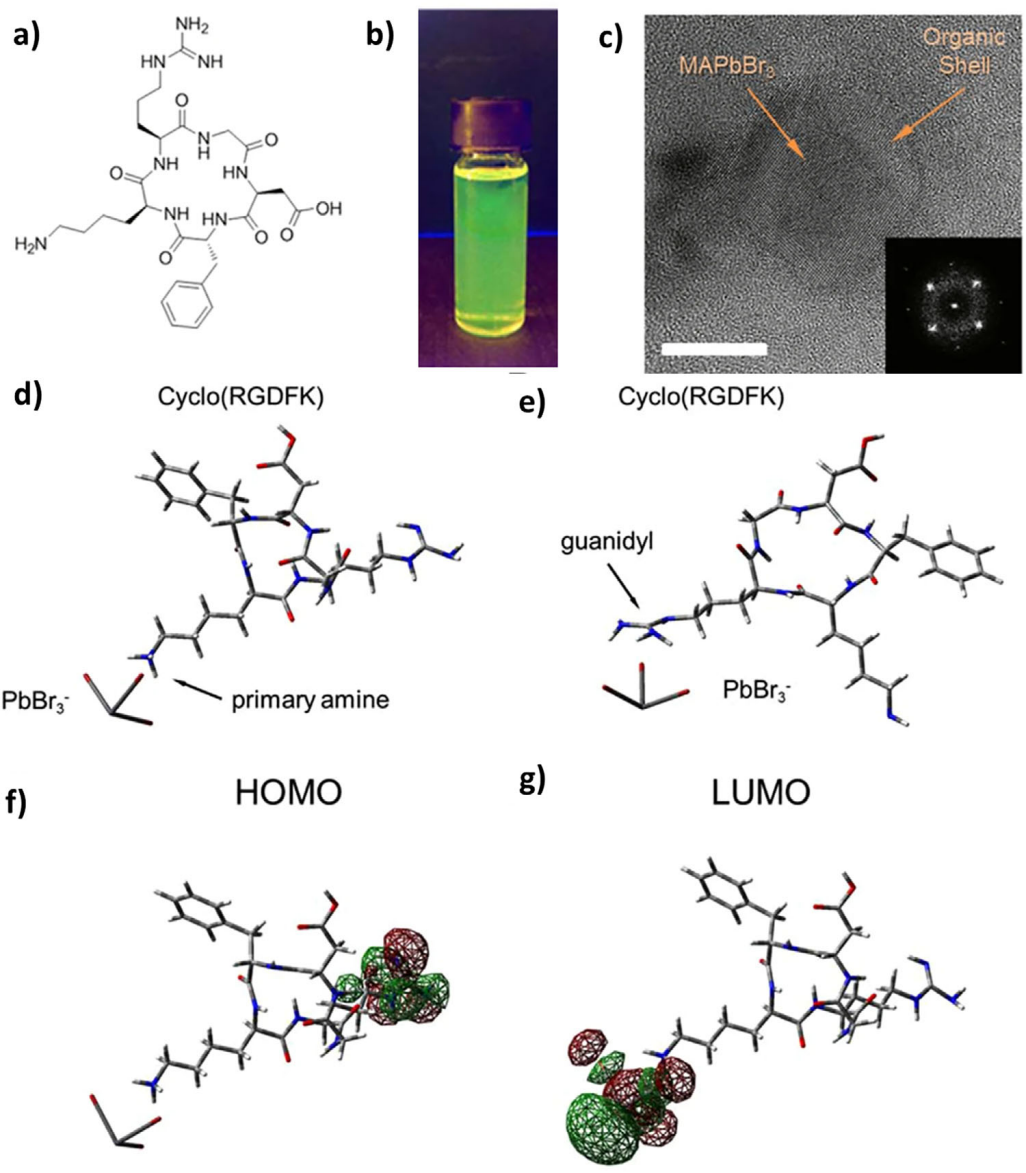
a) Chemical structure of cyclic pentapeptide. b) digital image of cyclic pentapeptide passivated MAPbBr_3_ NCs under UV‐light. c) TEM images of corresponding PNPs with FFT pattern inset (scale bar 5 nm). d‐g) B3LYP/6‐311+G* Optimized geometries of the PbBr_3_^−^/Cyclo(RGDFK) precursor complexes, (d) where the peptide is coordinated to the PbBr_3_^−^ with the primary amine group and (e) where the coordination bond is formed between PbBr_3_^−^ and guanidyl group; Isosurfaces of electron density distribution at HOMO (f) LUMO (g) electronic orbitals in the complex (d). Reproduced under the terms of CC BY 4.0.^[^ ^79^ ^]^ Copyright 2019, The Authors, published by Springer Nature.

Further, they demonstrated the generality of peptide capping by extending the synthesis from MAPbBr_3_ to CsPbBr_3_. Galvanized from the previous reports of higher solution and conformation stability of cyclic peptides than their linear counterparts, a cyclic pentapeptide (RGDFK) (**Figure** **9**a,b) was used as capping ligand for MAPbBr_3_ nanocrystals with an average size distribution of 6 ± 2 nm (Figure 9c) and photoluminescence quantum yield of 20%.^[^ ^79^ ^]^ It has been reported that the optoelectronic properties and defect formations are directly related to the coordination of complex formations or interactions that occur in the precursor solution. Since the PNC formation is ensured through the termination of perovskite crystallization by the amino groups in the precursor solution. Therefore, understanding the kinetics of precursor solutions is vital for peptide‐PNC research. Addressing this concern, quantum chemical calculations were made and a complex formation was proposed in which cyclic peptide coordinated to the PbBr_3_^−^ through the α‐amino group controls the formation of nanoparticles. It was shown that the guanidyl group, another putative cyclic peptide coordinating site, extends directly outward from the PNC surface. (Figure 9d‐g). The other potential coordinating site of a cyclic peptide, i.e., guanidyl group, directed outwards from the PNC surface is hypothesized as the cause of the decreased PLQY due to charge transfer from perovskite to the peptide shell.

**Figure 10:**
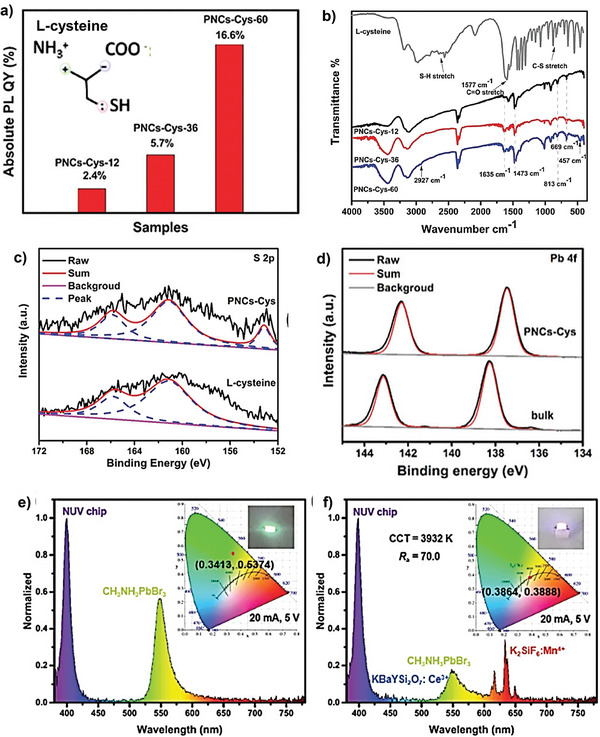
a) Absolute PLQYs for PNCs‐Cys prepared with different aging times, inset shows the chemical structure of L‐cysteine. b) FTIR spectrum of pure L‐cysteine (top, grey) and PNCs‐Cys aged for different times (12, 36, and 60 h). c) XPS spectra corresponding to S2p d) Pb 4f and of PNCs‐Cys a bulk materials. e) Emission spectra corresponding to PeLEDs and f) WLEDs encapsulated with PNC‐Cys‐60; inset: photographs of the CIE color coordinates and related LEDs. Reproduced with permission.^[^ ^80^ ^]^ Copyright 2018, Royal Society of Chemistry.

The synergic effect of amino, carboxylic, and sulfhydryl groups in a trifunctional L‐Cysteine amino acid led to strong interaction with the PNCs which effectively passivate the surface defect for long‐term structural and chemical stability in protic solvents and high PLQY (**Figure** **10**a) with a long PL lifetime of 642 ns was achieved for PNCs‐Cys‐60.^[^ ^80^ ^]^ The better stability of PNCs‐Cys‐60 in protic solvents was attributed to improved surface passivation through crosslinking from the L‐cys. The role of L‐cysteine as a surface passivating ligand was established by FTIR spectroscopy suggesting that the carboxyl group participates in passivation through a relatively strong interaction and C═O stretching bond shifts to high wavenumber(Figure 10b). Besides, coordination bonding between the sulfhydryl group and Pb^2+^ was also hypothesized in the case of PNCs‐Cys which is further corroborated by XPS measurements (Figure 10c,d). These cysteine‐capped PNCs exhibit strong green fluorescence and were explored for WLEDs and PeLEDs (Figure 10e,f) and achieved competitive performance.

**Figure 11:**
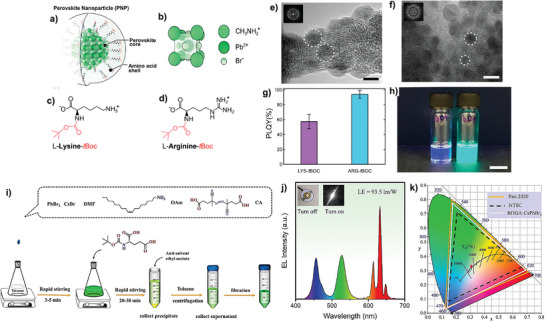
MAPbBr_3_ PNC capped with peptide ligands: a) schematic representation of PNC. b) crystalline structure of MAPbBr_3_, the core material of PNC. c‐d) Chemical structures of (c) Lysine (LYS) and (d) arginine (ARG) modified with tBOC group at their α‐amino group position (highlighted in red) to promote selective bonding to the PNP perovskite core LYS‐tBOC (scale bar = 5 nm) and (d) ARG‐tBOC (scale bar = 10 nm). The images clearly show the formation of nanoparticles (highlighted with dashed lines) with an average diameter of d(LYS‐tBoc) = 4.4 ± 0.3 nm and d(ARG‐tBoc) = 5.7 ±0.3 nm. g) PLQY results for LYS‐tBoc and ARG‐tBoc samples. h) Photographs of LYS‐tBoc and ARG‐tBoc PNC suspensions under UV illumination show bright blue and cyan luminescence visible to the naked eye (scale bar = 1 cm). Reproduced with permission.^[^ ^81^ ^]^ Copyright 2019, American Chemical Society. i) Schematic illustration of the ligand post‐treatment and purification process. j) The EL spectrum of WLED device. The inset shows the as‐fabricated WLED device without/with an operating current of 20 mA at 2.6 V. (k) The color coordinate and color gamut of the WLED (white line) compared to an NTSC standard (black dashed line) and the Rec. 2020 standards (yellowish‐brown line). Reproduced with permission.^[^ ^77^ ^]^ Copyright 2022, Elsevier.

In another work, L‐lysine and L‐arginine assisted MAPbBr_3_ nanocrystals showed PLQY of ≈100%.^[^ ^81^ ^]^ The higher PLQY was achieved via a preferential ligand orientation where the BoC as the protecting group blocked the α‐amino group coordination. Thereby forcing the remaining positive charged functional groups, i.e., amine (L‐lysine) and guanidine (L‐arginine), to coordinate with the anionic defects, which in turn delivers the uniform particle size distribution along with the higher PLQY in comparison with the unprotected α‐amino group scenario (**Figure** **11**a‐h). This also induced the possibility of further functionalization of the α‐amino end, which needs to be explored. Similarly, various reports appear with the PLQY and structural, thermal, and chemical stability enhancements with peptide‐like ligand engineering in PNCs.^[^ ^82^, ^83^, ^84^ ^]^

A multidentate ligand post‐treatment exchange strategy (Figure 11i) was adopted to attain brightly luminescent CsPbBr_3_ NCs for wide color gamut display applications (Figure 11j‐k). The multidentate ligand, Boc‐D‐Glutamic acid (BDGA) allows the PLQY of BDGA@CsPbBr_3_ nanocrystals to reach close to unity (due to passivation of the surface defects) and improved colloidal, thermal treatments, and UV irradiation stability.^[^ ^77^ ^]^

The smaller dielectric constants of organic ligands limit the charge transfer properties of PNCs and thus restrict their applications in electronic devices other than LEDs.^[^ ^85^ ^]^ Capitalizing on the prior reports that the peptide nucleic acids (PNAs) have much higher dielectric constants than the typical organic molecules.^[^ ^86^ ^]^ Pioneering work on PNA‐capped PNCs was suggested^[^ ^87^ ^]^ and the authors opted for a thymine‐based peptide nucleic acid monomer and trimer as the capping ligand for MAPbBr_3_ nanocrystals. In the competition between the thymine group and the primary‐amine group for coordinating with the PNC surface, the unprotected primary‐amine group showed a high preference. The uncoordinated thymine group reduced the PLQY. However, akin to Deoxyribonucleic acid, PNA is known for charge transfer between nucleic acids.^[^ ^88^ ^]^

It is known that the unprotected thymine group forms hydrogen bonds with their complementary pair of adenine nucleic acids. The authors supplemented the PNC colloidal solution with adenine nucleic acid to make the scenario plausible. The charge transfer between adenine and the PNC surface‐modified thymine group was evidenced by the observed PL quenching. The authors proposed the suitability of peptide‐modified PNCs as nucleic acid sensors due to the PL quenching, and they reached a detection limit of 2 ppm, which is similar to other reported nucleic acid sensors.^[^ ^89^ ^]^

### 4.2 The Use of Short Peptides in Perovskite Solar Cells

#### 4.2.1 In Bulk Perovskite

The photovoltaic performance and environmental stability of solar cells are compromised by electronic imperfections found at the surfaces and grain boundaries of perovskite crystals. Despite the exceptional optoelectronic properties of these soft ionic materials, they are unstable in real operating atmospheric conditions such as air and moisture due to internal ion migration that is further sped up by temperature changes, voltage bias, and light.^[^ ^90^ ^]^ Several strategies have been implemented to stabilization of hybrid perovskite materials and devices to improve the efficiency and environmental stability of PSCs, including composition engineering, interfacial modification,^[^ ^91^, ^92^ ^]^ additivitization,^[^ ^82^, ^93^ ^]^ and passivation using organic molecules^[^ ^94^, ^95^ ^]^ or polymers with different functional groups.^[^ ^86^, ^96^ ^]^

It has been shown that strong hydrogen bond donors, including ammonium (NH_3_^+^) groups, can interact with the A‐cation vacancies on the surface of hybrid perovskites by Hydrogen bonding.^[^ ^97^, ^98^ ^]^ This could lead to morphological alterations, the passivation of anionic defects (halide ions), or an increase in environmental stability brought about by introducing resistance to moisture and oxygen. These effects can be strengthened when multiple functional groups are used in the systems. These groups, for instance, engage with the perovskite surface and simultaneously passivate perovskite defects or crosslink perovskite grains.

In this regard, peptide‐like molecules containing amine (─NH_2_) and carboxyl (─COOH) groups were found to be a good candidate due to their multiple functionalities, including stabilizing the perovskite phase, repairing defects, and regulating morphology.^[^ ^99^, ^100^, ^101^, ^102^ ^]^

For example, as a “molecular bridge,” the amino acid lysine, which has two NH^3+^ groups, is integrated into the hybrid unit cell of MAPbBr_3_ by replacing two methylammonium ions. This inclusion causes the host's lattice parameter to decrease while also increasing its bandgap and causing discernible morphological alterations. Moreover, MAPbBr_3_ exhibits a shift in its cubic‐to‐tetragonal phase change temperature and a significant decrease in its thermal expansion coefficient. Furthermore, adding lysine significantly improves the stability of MAPbBr_3_ perovskite under humid conditions. When Lys is added during slow growth, the MAPbBr_3_ dissolution rate is reduced by ≈40%.^[^ ^101^ ^]^

Hence, passivating the defects of perovskites by adding peptides to the perovskite precursors or depositing them on top of the surface improved photovoltaic performance and stability under humid conditions.^[^ ^103^, ^104^, ^105^, ^106^ ^]^ For example, butyl phosphonic acid 4‐ammonium chloride (4‐ABPACl) and 5‐amino valeric acid (5‐AVA) were used as molecular additives to cross‐link the perovskite grains via hydrogen bond with phosphonic acid and a carboxylic acid, respectively. Further, the existence of these molecular crosslinkers at the grain boundaries prohibited water from piercing the perovskite coating. The fabricated mesoporous n‐i‐p solar cells using mixed‐cation perovskite (5‐AVA)_x_(MA)_1‐x_PbI_3_ templated with 5‐AVA attained a certified power conversion efficiency of 12.8% and maintained stability for more than 1000 h in ambient air under continuous sunlight.^[^ ^107^ ^]^

A new functionalized phenyl alanine amino‐acids‐based molecule, known as D‐4‐tert‐butyl phenylalanine is developed to passivate the perovskite surface and grain boundaries, where ‐NH_2_ and –COOH groups can passivate the charged defects via electrostatic interactions and aromatic rings suppressed the neutral iodine‐related defects by reducing the molecular iodine.^[^ ^103^ ^]^ Using D‐4‐tert‐butylphenylalanine, a power conversion efficiency (PCE) of 21.4% was achieved in p‐i‐n solar cells. With an optical bandgap of 1.57 eV for the used perovskite, the maximum open‐circuit voltage (*V*_OC_) of these devices reaches 1.23 V, exhibiting a small *V*_OC_ deficit of 0.34 V.

**Figure 12:**
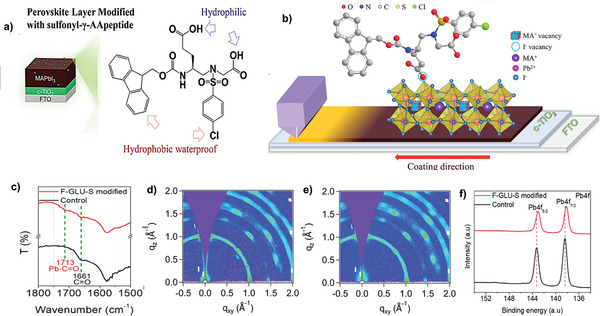
Schematic illustration of a) perovskite film passivation by F‐GLU‐S and molecular structure of F‐GLU‐S. b) interaction of F‐GLU‐S with MAPbI_3_ perovskite; Characterization of the interaction between MAPbI_3_ and F‐GLU‐S. c) FTIR spectra of the control and 2 mM F‐GLU‐S modified MAPbI_3_. d) GIWAXS of the control perovskite. e) GIWAXS of the modified perovskite, the small spot at 0.895 Å for the control sample corresponds to the red rectangle which clearly showed the presence of uncoordinated Pb^2+^ in the control perovskite. f) High‐resolution XPS spectrum for the Pb 4f of the control and 2 mM F‐GLU‐S modified MAPbI_3_ films. g) Schematic illustration of the fabricated device with a structure of FTO/c‐TiO_2_/MAPbI_3_ with and without 2 mM F‐GLU‐S modification/Spiro‐OMeTAD/Au. h) The champion device performance using the control and 2 mM F‐GLU‐S modified MAPbI_3_. Reproduced with permission.^[^ ^108^ ^]^ Copyright 2023, Elsevier.

In another study, a combination of theoretical and experimental research was used to investigate several kinds of natural amino acids (NAAs)^[^ ^100^ ^]^ as passivating agents for the fabrication of MAPbI_3_ based PSCs. The various molecular interaction strengths have a direct correlation with the optoelectronic properties and photovoltaic performance of NAA‐modified MAPbI_3_ films. Because of the strong coordination with the uncoordinated Pb^2+^, arginine (Arg) functionalized with a guanidine end group demonstrates the optimized passivation effect. This effectively lowers the antisite Pb_I_ deep‐level defects. Subsequently, the blade‐coated Arg‐passivated PSCs yield a high PCE of 20.49% and a *V*_OC_ of 1.17 V, which is ≈100 mV higher than that of pristine devices. The significance of these results was highlighted by a large‐area solar module (10.08 cm^2^) that showed a very high efficiency of 15.65% with minimal *V*_OC_ losses. Recently, large area slot‐die coated perovskite solar cells in the n‐i‐p configuration were fabricated using artificial peptide sulfonyl‐γ‐AA peptide (F‐GLU‐S) to modify the slot‐die‐coated perovskite surface, grain boundaries, and passivating the defects (**Figure** **12**a,b).^[^ ^108^ ^]^ The multi‐functional short AA peptide containing carbonyl, carboxyl, sulfonyl, benzene, and chloro groups is found to interact strongly with the perovskite layer and can repair the halide vacancies and uncoordinated Pb^2+^ ions, which is validated by FTIR, GIWAXS and XPS characterizations (Figure 12c‐f). The FTIR characterization unravels the strong interaction between peptide and perovskite through the carbonyl group (Pb─C═O). This interaction successfully reduced the uncoordinated Pb^2+^ ions, as demonstrated by 2D GIWAXS and XPS peak shifts. Furthermore, I^−^ vacancies were successfully restored because of the F‐GLU‐S's interaction with the perovskite by hydrogen bonding the (N–H…Cl). The modified device's performance improved significantly in terms of both *V*_OC_ and FF, indicating that non‐radiative recombination was effectively suppressed. Consequently, devices based on F‐GLU‐S modified slot‐die coated MAPbI_3_ showed exceptional PCE of 21.44%. Furthermore, because of its hydrophobic properties and the ability for defect healing, F‐GLU‐S passivation prevented moisture and oxygen from penetrating, and the solar cell device showed high moisture stability, as suggested by the increased water contact angle. The effect of F‐GLU‐S modification on the long‐term device operational stability of PSCs was also studied by keeping the control and modified devices for one month at relative humidity (RH) of 50–60% in air. The *V*_oc_ was unaffected by the moisture, suggesting that the TiO_2_ layer was extremely compact, however, FF and *J*_sc_, both declined rapidly of the control devices. It is noteworthy that the F‐GLU‐S modified devices demonstrated remarkable operational stability, maintaining ≈92% of their original performance after 720 h.

#### 4.2.2 Peptides at Charge Transporting Interface

Besides, the imperfection at surfaces and grain boundaries of perovskite crystals, controlling the charge transfer characteristics at the interface between layers, i.e., perovskite and electron (hole) transporting layer is another challenge in the fabrication of efficient PSCs. Unidirectional charge transfer from perovskite to the electron or hole transporting layer is preferred and recommended with the low charge transfer resistance. The intensity of PL in a perovskite film is significantly reduced (i.e., PL quenching) when there is a significant charge transfer from the perovskite layer to the subsequent ETL or HTL. Thus, straightforward measurements of steady‐state PL enable the evaluation of the interfacial charge transfer property. Additionally, charge transfer resistance for PSCs can be quantitatively characterized using electrochemical impedance spectroscopy (EIS), which demonstrates less charge transfer resistance at the ETL/perovskite or perovskite/HTL interface and can be further corroborated by the lower steady‐state photoluminescence (PL) intensity and shorter perovskite PL lifetime.

**Figure 13:**
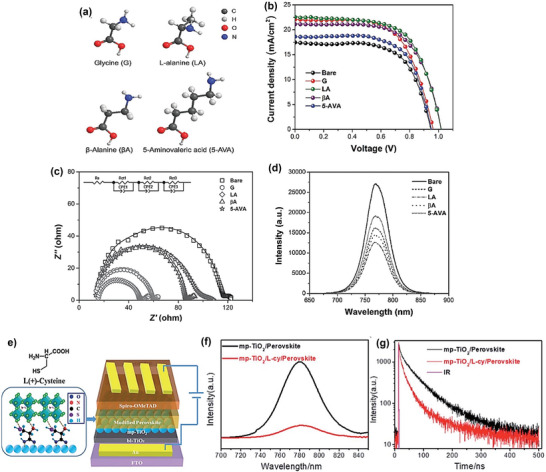
a) Chemical structure of amino acids. b) *J–V* curves of amino‐acid‐modified PSCs measured by backward scan with 10 mV voltage steps and 200 ms delay time under standard AM 1.5 illumination (100 mW cm^−2^. c) Nyquist plots of EIS for the indicated amino‐acid‐modified PSCs measured under constant light illumination of 100 mW cm^−2^. d) PL spectra of the perovskite films formed on bare m‐TiO_2_ and those modified with the indicated amino acids. Reproduced with permission.^[^ ^99^ ^]^ Copyright 2017, John Wiley and Sons. e) The schematic structure of the PSC was modified using L‐cy. f) steady‐state PL spectra. g) time‐resolved spectra of the perovskite films on bare mp‐TiO_2_ and on the L‐cy modified mp‐TiO_2_. Reproduced with permission.^[^ ^109^ ^]^ Copyright 2020, Royal Society of Chemistry.

Different amino acids (**Figure** **13**a) have been used to modify an electron‐transporting layer (TiO_2_), which influences the efficiency of charge transfer at the TiO_2_/CH_3_NH_3_PbI_3_ interface in PSC. The l‐alanine‐modified cell shows the best PV performance (Figure 13b) with a 30% improvement compared to the reference cell.^[^ ^99^ ^]^ Following amino acid modification, electrochemical impedance spectroscopy (EIS) (Figure 13c) demonstrates less charge transfer resistance at the TiO_2_/CH_3_NH_3_PbI_3_ interface, which is further corroborated by the lower steady‐state photoluminescence (PL) intensity (Figure 13d) and shorter perovskite PL lifetime. In another report, the interface between mesoporous TiO_2_ (mp‐TiO_2_) and perovskite is modified using L‐cysteine (L‐cy), a typical trifunctional amino acid. (Figure 13e) and the PCE rises from 11.5% to 14.4% after modification. Steady‐state and time‐resolved fluorescence spectra experiments (Figure 13f,g) demonstrate that the interactions between the functional groups (‐COOH, ‐NH_2,_ and ‐SH) of L‐cy and Ti^4+^ ions in mp‐TiO_2_ and unsaturated Pb^2+^ and I^−^ ions in the perovskite layer improve the electron injection from perovskite to mp TiO_2_.^[^ ^109^ ^]^

### 4.3 Future Strategies to Implement Short‐Peptides in Halide Perovskites

As of now, short peptides have been explored as surface‐capping ligands or passivators for perovskite solar cells and LEDs. However, the use of aromatic short‐peptide is very limited and can be further explored in many different ways for halide perovskite‐based optoelectronics and biomedical and photochemical applications. Future Prospects are suggested for the creation of the perovskite‐peptide system.

**Figure 14:**
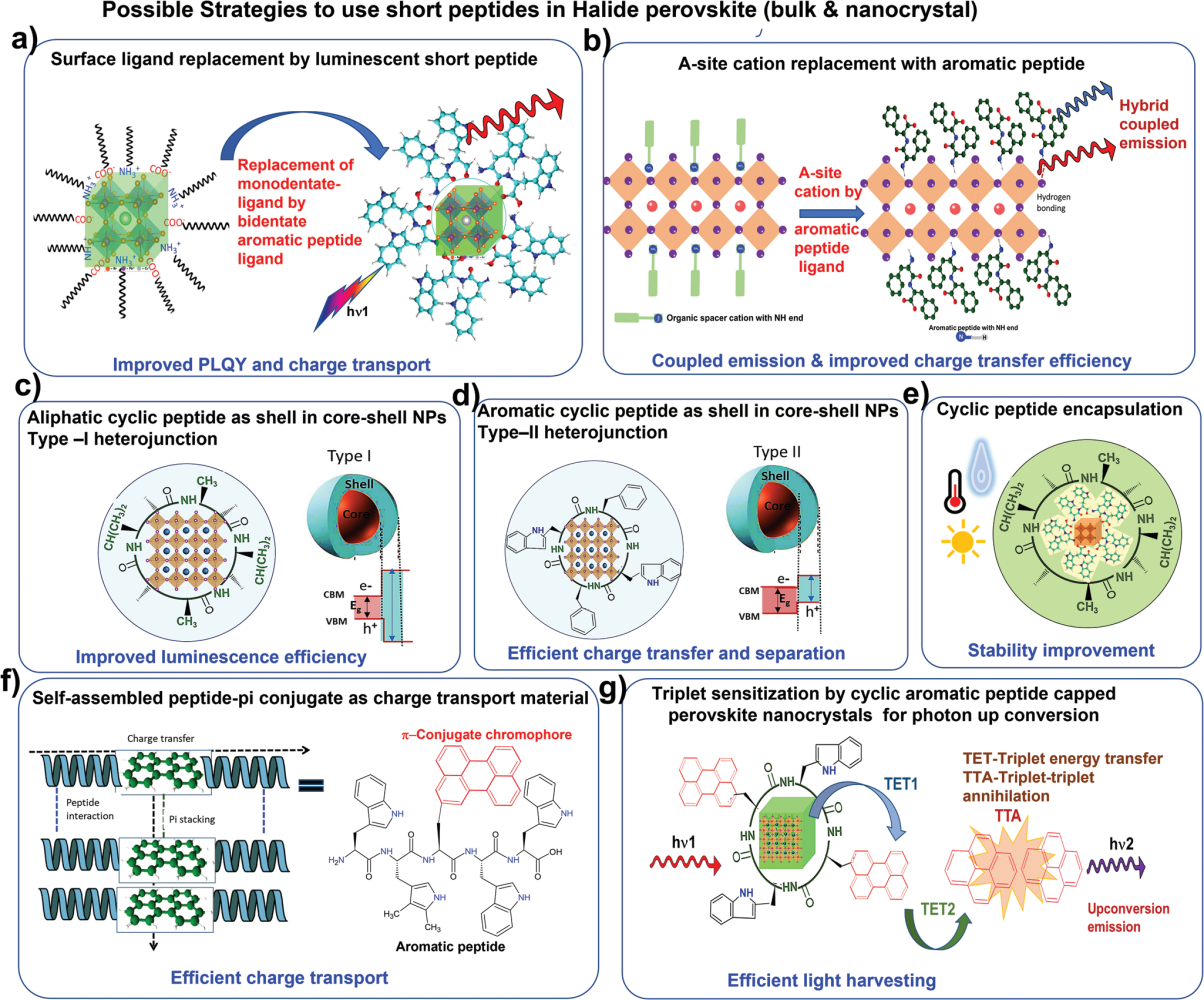
Conceptual illustrations to use short peptides in halide perovskite (bulk & nanocrystal).(a) Surface ligand replacement by luminescent short peptide, (b) A‐site cation replacement with aromatic peptide, (c)Aliphatic cyclic peptide as shell in core‐shell NPs Type –I heterojunction, (d) Aromatic cyclic peptide as shell in core‐shell NPs Type–II heterojunction, (e) Cyclic peptide encapsulation, (f) Self‐assembled peptide‐pi conjugate as charge transport material, (g) Triplet sensitization by cyclic Aromatic peptide capped perovskite nanocrystals for photon up conversion.

Bulk and nanostructures halide perovskite with organic ligands have been explored to passivate the surface of the perovskite films.^[^ ^110^ ^]^ Similarly, aromatic peptides with bidentate ligands ─COOH and ─NH_2_ group can self‐assemble at the surfaces and grain boundaries to passivate the under‐coordinate Pb^2+^ and halide vacancies in bulk and nanocrystals and improved PL emission with high quantum yield and carrier lifetime is expected. Moreover, charge transport properties will be improved due to aromatic conjugated moieties and a dual peak emission from hybrid perovskite and surface‐attached luminescent aromatic peptide might be expected (**Figure** **14**a). Besides, surface passivation, these short‐aromatic peptides containing ammonium groups can be also incorporated as A site organic luminescent cation in the layered or 2D halide perovskites (Figure 14b). The addition of the aromatic cations in the layered 2D perovskite lattice increases the excited state pathways and these hybrid peptide‐perovskite systems are easy to prepare using facile solution‐processable techniques.^[^ ^111^, ^112^ ^]^ Effective transfer of electrons from the inorganic layer to the organic moiety in a 2D perovskite structure allows the organic molecules to show phosphorescence at room temperature.^[^ ^112b^ ^]^ A dual emission center: which arises from quenched excitonic emission from the inorganic layer and phosphorescence from the organic peptide moiety can also be generated as a result of efficient triplet energy transfer (TET). Besides, the coupled emission color can be tweaked by adjusting the triplet energy levels, the functioning of the organic molecules, and the degree of close contact between them and the inorganic layer. Conversely, in contrast to 3D systems, the sensitized triplets in 2D perovskites can function as sources of long‐lived radiative emission even in an ambient atmosphere. Due to the layered structure, they are intrinsically shielded from oxygen by the stiff framework and local surroundings.^[^ ^112b^ ^]^

Producing core−shell heterostructures of perovskite NCs as core and linear or cyclic aromatic peptide as shell is another possible strategy to increase the excited state pathways and improve the charge transfer^[^ ^79^ ^]^ and chemical & structural stability of colloidal semiconductor QDs.

Cyclic aliphatic or aromatic short peptides can be used as a template to grow PNCs. The presence of aromatic moieties in cyclic peptides will lead to the narrowing of the band gap compared to the aliphatic cyclic peptide. Further through side group engineering on the cyclic peptide, either by adding an electron‐withdrawing or electron‐donating group, the interfacial energy level can be aligned for the PNCs, and a type I (nested) or type II (staggered) heterojunction can be constructed. Thus, a demonstration of structural templating of the organic layer can be made possible through side‐group engineering (Figure 14c,d).Moreover, to avoid self‐aggregation of the cyclic peptide ligand during the crystallization process, sterically demanding methyl groups can be incorporated into the aromatic moieties to quench π−π interactions between peptides.

Besides core‐shells, these linear or cyclic peptides with long alkyl chains can be explored as a thick layer encapsulating shell to protect halide PNCs from environmental stress factors and enhance their moisture stability (Figure 14e). The hydrophobicity of peptides can be enhanced by using amino acid residue with a more hydrophobic side group (aromatic group) such as phenylalanine and leucine.

Self‐assembled aromatic short peptide structures can enhance electronic properties by enabling long‐range molecular ordering and by offering more tuning factors to regulate the characteristics of the devices. Thus, this aromatic peptide can be also used as charge transporting materials for perovskite optoelectronics due to their intrinsic semiconductor behavior which can be further conjugated with external functional moieties such as traditional organic semiconductors to create the hybrid bio‐self‐assembled supramolecular organic semiconductor with improved performance and open up a new venue for molecular electronics applications.^[^ ^25^ ^]^ Peptide functionalized with p‐type^[^ ^25^, ^111^ ^]^ and n‐type conjugated^[^ ^24^, ^112^ ^]^ molecular semiconductors have already been explored. Aromatic π‐conjugated units, such as thiophene, perylene diimides (PDIs), and naphthalene diimide (NDI), are ideal for supramolecular electronics and depending on their intrinsic n‐type or p‐type organic semiconducting properties that confer desirable charge transfer properties and their strong intermolecular aromatic–aromatic interactions that promote self‐assembly (Figure 14f).

Another possibility is to use these aromatic short‐peptides as triplet acceptors in the triplet−triplet annihilation‐based photon up‐conversion process(TTA‐UC),^[^ ^112c^ ^]^ where the 3D‐halide PNC works as triplet sensitizer to transfer triplet energy to surface attached organic short‐peptide containing a small conjugated unit such as pyrene etc., which, in turn transmit the triplet energy to free organic molecules in the solution.The bandgap of the PNC must be tuned in such a way that the energy level must match with the acceptor triplet (Figure 14g).

## 5 Summary and Future Outlook

We delve into the use of biocompatible short‐peptides in the field of halide perovskites (bulk and nanocrystals) for solar cells, light‐emitting diodes, and sensing applications, including the self‐assembling characteristics and inherent optoelectronic properties. The inherent energy transfer mechanisms in their self‐assemblies conferred a semiconductive property to the peptides and also resulted in elevated PLQY values for the peptides and their self‐assemblies. The structural diversity, including π‐π aromatic conjugated moieties, hydrogen bonding, and cyclic arrangements, gave light‐emitting self‐assemblies with higher PLQY values. We elucidated the electron transfer mechanisms within peptide self‐assemblies, that entail length‐dependent electron tunneling and structure‐dependent electron hopping pathways. In longer peptides charge transport occurs through electron hopping, while in shorter peptide chains, it transpires via electron tunneling. The bidentate ligand characteristics of peptides with amine and carboxylic termini can serve as possible surface capping ligands by passivating both cationic and anionic defect sites. The perovskite nanocrystals synthesized with peptide capping ligands by the ligand‐assisted reprecipitation (LARP) technique showed high PLQYs and enhanced colloidal stability. Beyond the amine and carboxylic termini, additional functionalization using sulfhydryl groups, suggests that multi‐functionalized peptides can be more effective in passivating the defects, and thus improving the colloidal stability. Besides the exploration of perovskite nanocrystals for LED applications, the short‐peptides identify prospects in low‐bandgap halide perovskites for application in perovskite solar cells. In this context, short‐peptide‐with one or two amino acid chains entailing amine and carboxyl groups shows improved photovoltaic properties by stabilizing the perovskite phase, repairing defects, and modulating shape. The interactions in devices with short‐peptides and halide perovskites can take place in three different ways: additivisation, passivation of the perovskite‐hole transport layer (HTL) interface, and passivation of the electron transport layer (ETL)‐perovskite interface. The short‐peptide additivisation mitigates electronic defects and minimizes *V*_OC_ deficit, whereas the passivation on the perovskite surface enhances hydrophobicity. Perovskite interfacial passivation by peptide facilitates effective charge transfer between the perovskite layer and the charge transport layer.

Despite the achievements and potential of peptides in optoelectronic devices, the peptide‐perovskite combination presently encounters several challenges. Primarily, the peptide‐perovskite nanocrystals investigations have predominantly concentrated on theLARP synthesis method, whereas the promise of the sophisticated hot‐injection technique remains unexamined. Second, the lack of understanding of potential competition between inter‐ and intra‐charge transfer mechanisms involving peptides and perovskites poses challenges in the rational design and selection of peptides, resulting in the examination of only a limited subset from the extensive library of short peptides. Third, lead toxicity in perovskite may inhibit the biocompatibility of peptide‐perovskite systems.

The biocompatibility, inherent semiconductive properties, and extensive emission spectrum from visible to near‐infrared enable these supramolecular structures to be investigated in optoelectronics and in vivo bioimaging applications, thereby opening a new pathway into the realm of bioelectronics. Besides, F_moc_ and *t*_Boc_ as protecting functional groups, the other functional group to functionalize the α‐amino end, needs to be explored. The suitability of these self‐assemblies for diverse device applications and integration of the perovskite nanocrystals into biological devices needs further investigation. In addition to the exploitation of short‐peptide functions in halide perovskite bulk and nanocrystal systems, uncovering the mechanistic inside of the perovskite‐peptide system is paramount. A Tryptophan (W)‐based aromatic peptide self‐assembling platform with high mechanical stiffness and intrinsic optoelectronic properties can be suitable for solar cells, field effect transistors, and bioelectronics applications. Directional approaches are essential to create rational peptides that can surmount existing limitations on the regulation of self‐assembled nanofibrillar structures tailored to specific environmental conditions, thus enhancing bioelectronics performance. Investigating the unique self‐assembling properties of short, customized peptides in conjunction with semiconducting lead‐free perovskite, where the binding and coordination of metal ions (e.g., Sn^2+,^ Ge^2+^) are critical, for substantial progress. Understanding the vast array of possible combinations necessitates the use of machine learning and artificial intelligence capabilities.  Obstacles remain to ensure the secure and extensive application of peptide self‐assemblies as semiconductors, including elucidating the nanostructure‐property relationships, deciphering the molecular mechanisms that govern physicochemical properties, and regulating the semiconductivity to facilitate the transition from scientific research to industrial applications.″

## Conflict of Interest

The authors declare no conflict of interest.
